# Effects of Sodium Hypochlorite Bleaching on the Quality and Safety of Basa (*Pangasius bocourti*) Fish Maw: Texture, Collagen Structure, and Semicarbazide Formation

**DOI:** 10.3390/foods15061001

**Published:** 2026-03-12

**Authors:** Honglin Zhang, Nan Pan, Xiaoyan Wang, Xiaoting Chen, Shuji Liu, Yongchang Su, Zhiyu Liu

**Affiliations:** 1 Institute of Postharvest Technology of Agricultural Products, College of Food Science, Fujian Agriculture and Forestry University, Fuzhou 350002, China; 13850415648@163.com (H.Z.); 17793478960@163.com (X.W.); 2 Key Laboratory of Cultivation and High-Value Utilization of Marine Organisms in Fujian Province, National Research and Development Center for Marine Fish Processing (Xiamen), Fisheries Research Institute of Fujian, No. 7, Haishan Road, Huli District, Xiamen 361013, China; npan01@qub.ac.uk (N.P.); xtchen@jmu.edu.cn (X.C.); cute506636@163.com (S.L.)

**Keywords:** fish maw, semicarbazide, oxidative bleaching, untargeted metabolomics, food safety

## Abstract

This study aimed to investigate the effects of sodium hypochlorite (NaClO) bleaching on the quality of Basa (*Pangasius bocourti*) fish maw (BFM) and the formation of semicarbazide (SEM). Production of SEM increased (*p* < 0.05) when NaClO concentration, soaking temperature, or duration were increased. Notably, increasing NaClO solution pH also enhanced SEM formation. Soaking BFM in NaClO with available chlorine concentrations of 500, 700, and 1000 mg/L generated 0.05, 0.07, and 0.09 μg/kg SEM at pH 3 compared to 0.70, 1.19, and 2.34 μg/kg SEM at pH 11, respectively. NaClO improved BFM texture by creating a tight, fibrous structure, but also damaged the secondary structure and α-chains of collagen. Untargeted metabolomics showed that NaClO treatment significantly upregulated lipid metabolism pathways (biosynthesis of unsaturated fatty acids, linoleic acid metabolism, and glycerophospholipid metabolism) and elevated degradation of arginine, proline, and urocanic acid. This was associated with the accumulation of nitrogen-containing precursors in the urea cycle, which then reacted with NaClO, generating substantial SEM. Controlled SEM-generating reactions experiments confirmed that SEM was produced from reaction of urea and NaClO. This study elucidates the mechanism of SEM formation and identifies key factors influencing SEM levels, thereby providing a theoretical foundation for safe processing and quality control of fish maw.

## 1. Introduction

Fish maw (dried swim bladder) is a highly popular traditional tonic food in China and Southeast Asia due to its low fat content and high nutritional value [1]. It is rich in amino acids, such as proline (Pro), glycine (Gly), and arginine (Arg), that are known to regulate blood glucose levels and promote tissue repair [2]. Collagen peptides derived from fish maw are associated with anti-aging, antioxidant, and anti-tumor effects [1,3]. The market for fish maw has considerable potential for development because of these functional properties and its nutritional value [4].

In response to growing consumer demand for convenient and nutritious foods, the food industry has developed a range of ready-to-eat fish maw products. Canned ready-to-eat fish maw has the largest market share, being most popular with consumers [5]. The raw material for fish maw is primarily the swim bladders of marine fish, such as large yellow croaker, crimson snapper, and cod. The market for freshwater fish such as basa (*Pangasius bocourti*) has expanded in recent years [6]. A diverse diet, ease of cultivation, rapid growth, large size, high yield, and low farming costs have contributed to basa becoming an important freshwater aquaculture species in Southeast Asian countries that is widely used in the production of ready-to-eat fish maw [6]. Basa was selected for this study due to its growing commercial importance in the ready-to-eat fish maw sector. However, there is a particular technological challenge in the processing and storage of canned ready-to-eat fish maw—deterioration of texture that can manifest as decreased hardness and chewiness, increased stickiness, and, in severe cases, structural disintegration of the product. Some manufacturers resort to illegal or excessive use of chemical bleaching agents to enhance the product’s heat resistance, chewiness, whiteness, and appearance [7]. This practice introduces potential food safety risks. Strong oxidizing bleaching agents, such as sodium hypochlorite (NaClO) and hydrogen peroxide (H_2_O_2_), are widely used in the fish maw rinsing process [8]. They sterilize the material, remove blood residues, and induce protein denaturation that improves the product’s heat resistance [9,10].

Semicarbazide (SEM), also known as carbamoyl hydrazine, is highly soluble in water (100.0 g/L at 20 °C) but insoluble in ether and ethanol [11]. It has long been recognized as a metabolite of the antibiotic nitrofurazone (NFZ) and is commonly used as a marker to monitor illegal use of NFZ in aquaculture [12]. While NFZ is rapidly metabolized in animals (having a half-life of only a few hours), SEM binds to proteins, forming stable complexes that persist and accumulate in tissues over extended periods [13]. SEM exhibits physiological toxicity [14], genotoxicity [15], and carcinogenic effects [16]. Residual SEM in animal-derived products can be transferred to humans via the food chain, since the acidic gastric fluid can release SEM from protein, allowing it to be absorbed [17]. Long-term intake of SEM threatens human health through carcinogenic and teratogenic effects, skin pigmentation, and kidney damage [18]. Studies have shown that the metabolic degradation of NFZ is not the sole source of SEM residues in aquatic products [19]. In 2004, Sarri et al. [20] first proposed the existence of endogenous SEM, and Hoenicke et al. [8] hypothesized that SEM is formed through the degradation of nitrogen-containing substances with amide or urea residues. Recent studies have suggested that SEM precursors include urea, hydrazine, arginine, histidine, citrulline, lysine, and glutamine [21,22]. Structural analyses indicate that both SEM and amino acids are nitrogen-rich compounds, leading to the hypothesis that SEM may be formed via the degradation of amino acids or other nitrogen-containing compounds [23].

Untargeted metabolomics is an unbiased analytical technique designed to comprehensively reveal changes in metabolites and associated pathways by identifying multiple small-molecule metabolites in biological samples such as blood, urine, and tissue [24]. Liquid chromatography–mass spectrometry (LC-MS) is widely used in untargeted metabolomics due to its high sensitivity, high resolution, and rapid detection capability [25]. It enables the simultaneous identification and quantification of multiple metabolites in complex biological matrices [26]. LC-MS-based untargeted metabolomics has been extensively applied in the study of metabolite changes during food processing [26] and could provide insights into such changes during fish maw bleaching. The potential of metabolomics as a robust, efficient, and sensitive analytical tool is widely recognized in food science [27]. It has been applied in research into the impacts of food on human health, in food processing and transformation studies, and in investigations into the mechanisms of SEM formation in aquaculture products [28].

NaClO is a broad-spectrum disinfectant widely used in food processing [29,30], particularly in fish [31] and ready-to-eat products [32], due to its potent bactericidal effects, strong oxidizing capacity, low cost, and minimal impact on nutritional and sensory quality [33]. However, safety concerns regarding its use have emerged, as high concentrations can generate disinfection by-products that pose potential health risks [34]. Notably, NaClO treatment has been reported to result in significant residues of SEM, a compound of regulatory concern, with levels exceeding the 1 µg/kg limit detected in various products [8,35,36]. According to information obtained from a fish maw processing facility, NaClO at concentrations of 700–1000 mg/L (available chlorine) is commonly used during processing. Therefore, in the present study, we employed NaClO solutions at concentrations ranging from 100 to 1000 mg/L to investigate the effects of both low and high concentrations on the quality of BFM and the formation of SEM. Despite the widespread application of NaClO in the seafood industry, its effects on SEM content and the overall quality attributes of flower gum products remain unexplored. Therefore, the present study aimed to investigate the impact of NaClO treatment on the quality and safety of flower gum, with a particular focus on SEM formation.

This study aims to bridge that knowledge gap by investigating how varying NaClO bleaching conditions impact both fish maw quality and SEM formation. To this end, we employ untargeted metabolomics to elucidate the underlying mechanisms. It also characterizes the collagen in fish maw and examines the effects of NaClO bleaching on its physicochemical properties. This study provides an essential foundation for the establishment of safer bleaching protocols that will ensure the food safety of fish maw and foster the sustainable development of its processing industry.

## 2. Materials and Methods

### 2.1. Materials and Reagents

Fresh basa (*Pangasius bocourti*) fish maw (BFM) (2.5 kg) was purchased from Gelan Ruike Co., Ltd. (Xiamen, China). It was cleaned to remove blood residues and trimmed of adherent fat and surface mucosa. SEM standard (purity > 99%) was purchased from Dr. Ehrenstorfer GmbH Co.(Augsburg, Germany), and SEM-^13^C-^15^N_2_ internal standard (purity > 99%) from Witega Co. (Berlin, Germany). Dimethyl sulfoxide and 2-nitrobenzaldehyde were purchased from Sigma-Aldrich Co. (St. Louis, MO, USA); ethyl acetate, formic acid, and methanol (HPLC grade) from Tedia Co. (Fairfield, OH, USA); and ammonium acetate (analytical grade), hydrochloric acid, sodium chloride, and dipotassium hydrogen phosphate from Sinopharm Chemical Reagent Co., Ltd. (Shanghai, China). Urea, amino acid standards, lecithin, and triglyceride were purchased from Yuanye Co., Ltd. (Shanghai, China), and 2× SDS-PAGE Loading Buffer and Feto SDS-PAGE Staining Buffer from Affinibody LifeScience (Wuhan, China). The two-color pre-stained protein marker (WJ102, 10–250 kDa) was purchased from Epizyme Biotech Co., Ltd. (Shanghai, China)

### 2.2. Sample Preparation

To investigate the impact of NaClO bleaching parameters on formation of SEM, BFM samples were treated within a single-factor experimental design. Preliminary tests indicated that bleaching duration, temperature, and pH had significant effects at concentrations above 500 mg/L available chlorine provided by NaClO. Three concentrations (500, 700, and 1000 mg/L) were therefore selected for subsequent systematic tests. For each treatment, BFM samples (50 ± 2 g) were immersed at a constant solid-to-liquid ratio of 1:10 (m/v). NaClO Concentration: Samples were immersed for 12 h at 25 °C in solutions (pH 7) of 100, 300, 500, 700, or 1000 mg/L available chlorine. Bleaching Duration: Samples were treated for 1, 6, 9, 12, or 24 h at the three selected concentrations (pH 7, 25 °C). Bleaching Temperature: Samples were incubated for 12 h at 4, 15, 25, 35, or 45 °C at the three selected concentrations (pH 7). NaClO Solution pH: pH of the three selected concentrations was adjusted to 3, 5, 7, 9, or 11 (using 1 M HCl/NaOH) before immersing samples at 25 °C for 12 h. After each treatment, samples were rinsed with distilled water and dried at 40 °C for 10 h in a forced-air oven (BGZ-240; Boxun Co., Ltd., Shanghai, China).

To investigate the effects of low- and high-concentration NaClO treatments on the quality of BFM and the formation of SEM, untreated BFM samples were immersed at a solid-to-liquid ratio of 1:10. The treatments were as follows: a control group (CK) was soaked in deionized water; a low-concentration treatment group (LC) in 100 mg/L NaClO (pH 7); and a high-concentration treatment group (HC) in 1000 mg/L NaClO (pH 7). After 12 h at 25 °C, the samples were rinsed and dried as described above. Sample physicochemical and collagen properties were then characterized, and untargeted metabolomics was applied to elucidate SEM formation pathways.

### 2.3. HPLC-MS/MS Analysis of SEM

SEM concentrations in the BFM samples were determined using a TSQ Quantum Ultra triple quadrupole LC-MS/MS system (Thermo Fisher Scientific, Waltham, MA, USA) according to Yu et al. [10]. Treated BFM samples (2.00 ± 0.01 g) were mixed with 100 μL of SEM-^13^C-^15^N_2_ internal standard (50 μg/mL), 5 mL of hydrochloric acid (0.5 mol/L, pH 3.5), and 100 μL of 2-nitrobenzaldehyde (50 mmol/L: 378 mg dissolved in 5 mL dimethyl sulfoxide). Derivatization proceeded at 37 °C for 16 h on a rotating shaker in a light-proof water bath. Samples were then extracted with ethyl acetate [10]. Chromatographic separation employed a C18 column (Thermo, Waltham, MA, USA) and mobile phase consisting of (A) 0.02% formic acid in water containing 5 mmol/L ammonium acetate and (B) 0.02% formic acid in methanol. The gradient elution program applied a constant flow rate of 0.25 mL/min. The mass spectrometer operated in positive electrospray ionization (ESI+) mode with the following optimized fragmentation parameters: SEM transitions *m*/*z* 209 > 166 (collision energy 11 eV) and *m*/*z* 209 > 192 (CE 13 eV), and internal standard transition *m*/*z* 212 > 168 (CE 11 eV). The calibration curve exhibited good linearity with a regression equation of Y = 0.172X − 3.94 × 10^−2^ (R^2^ = 0.9997). The method exhibited a limit of detection of 0.5 μg/kg, a limit of quantification of 1.0 μg/kg, and recoveries ranging from 70% to 120% at spiking levels of 1.0–10.0 μg/kg.

### 2.4. Physicochemical Properties

#### 2.4.1. Determination of Texture and Shear Force

The texture profile (hardness and chewiness) and shear force of BFM samples were determined using a texture analyzer (TA.XT plus, Stable Micro Systems, Surrey, UK), following the method of Yin et al. [37]. Dry CK, LC, and HC BFM samples (50 g each) were steamed for 10 min and rehydrated in distilled water (1:10, *w*/*v*; 25 °C) until their weight reached four times the initial weight. Samples were then cut into blocks (2 cm × 2 cm × 1 cm). Texture profile analysis (TPA) used a P/36R cylindrical probe under the following settings: test speed, 1 mm/s; return speed, 1 mm/s; deformation, 50%; and trigger force, 10 g. Shear force was measured using an A/CKB probe with a test speed of 10 mm/s, post-test speed of 5 mm/s, trigger force of 15 g, and compression distance of 30 mm.

#### 2.4.2. Scanning Electron Microscopy

The BFM microstructure was examined using a SU8100 scanning electron microscope (Hitachi High-Tech Corp., Tokyo, Japan) according to the method of Yang, et al. [38]. BFM samples (0.5 cm × 0.5 cm) were fixed in 2.5% glutaraldehyde at 4 °C for 24 h, then dehydrated through a graded ethanol series (30%, 50%, 70%, 80%, 90%, 95%, and 100%, each step lasting 15 min). Samples were mounted using conductive adhesive on the stage of an ion sputter coater and sputter-coated with gold before observing under 5000× magnification.

### 2.5. Collagen Characterization

#### 2.5.1. Collagen Extraction and Purification

The entire process of collagen extraction from BFMs was conducted at 4 °C. Samples (20.0 ± 0.5 g) were cut into 0.5 cm × 0.5 cm pieces and soaked in 0.1 M sodium hydroxide at a ratio of 1:10 (*w*/*v*), stirring continuously for 48 h to remove non-collagenous proteins, with the alkaline solution being replaced every 6 h. Samples were then rinsed with deionized water until neutral pH was achieved. Acetic acid (0.5 M) was then added at a ratio of 1:10 (*w*/*v*) and extraction was performed at 4 °C for 24 h with constant stirring. This solution was filtered through two layers of gauze and saturated sodium chloride solution was added to the filtrate to precipitate collagen via salting-out. After standing overnight, the precipitate was recovered by centrifugation (8000 rpm for 10 min), redissolved in 0.5 M acetic acid at a ratio of 1:5 (*w*/*v*), and dialyzed against 0.1 M acetic acid for 24 h and then deionized water for 48 h, with the dialysate being changed twice daily [39]. The purified collagen was finally freeze-dried and stored.

#### 2.5.2. Determination of Collagen Content

Samples (0.2 ± 0.01 g) from the CK, LC, and HC BFM groups were placed in hydrolysis tubes, mixed with 2 mL of 6 M HCl, sealed, and digested in a 110 °C oven until no large visible fragments remained. After cooling, the hydrolysates were adjusted to pH 6–8 using 10 M NaOH and diluted to a final volume of 4 mL with distilled water [37]. The solutions were centrifuged at 10,000 rpm and 4 °C for 20 min (5810 R; Eppendorf, Hamburg, Germany), and the supernatants were recovered. The hydroxyproline (Hyp) content was determined according to the method described by Naveena et al. [40]. A standard calibration curve of known concentrations was used to quantify Hyp (y = 0.9967x − 0.1283, R^2^ = 0.9997). Collagen concentration was calculated by multiplying Hyp concentration by a factor of 7.14 [41].

#### 2.5.3. Fourier Transform Infrared (FTIR) Spectroscopy

Dry potassium bromide (KBr, 100 mg) and freeze-dried collagen samples (1 mg) were placed in an agate mortar and ground to a fine, homogeneous powder. The mixture was loaded into a pellet die and pressed manually to form a transparent pellet. FTIR spectra were acquired using an FTIR spectrometer (Nicolet Summit LITE; Thermo Fisher Scientific, Waltham, MA, USA) over the range 4000–400 cm^−1^ at a resolution of 4 cm^−1^ [42].

#### 2.5.4. SDS-PAGE Electrophoresis

Extracted collagen was dissolved in 0.5 M acetic acid, giving a 200 μg/mL solution that was mixed at a 1:1 (*v*/*v*) ratio with sample buffer (0.5 M Tris-HCl, pH 6.8, containing 4% SDS and 20% glycerol) and denatured in a 98 °C water bath for 5 min. A 20 μL aliquot of the denatured sample was loaded into each well of a 12% polyacrylamide separating gel [39]. Electrophoresis was performed in Tris-glycine-SDS running buffer at a constant voltage of 120 V until the dye front reached the bottom of the gel. SDS-PAGE Staining Buffer was then applied to the gel until protein bands were clearly visible. The gel was then destained with deionized water until the background became clear. Images of gels were digitized using a flatbed scanner (Epson Perfection V 700; Epson, Nagano, Japan) for further analysis.

### 2.6. Untargeted Metabolomics

#### 2.6.1. Sample and Quality Control Preparation

BFM samples (25 ± 1 mg) were homogenized in 500 μL of ice-cold extraction solvent (methanol:acetonitrile:water, 2:2:1, *v*/*v*) containing deuterated internal standards using a bead-beater (35 Hz, 4 min) followed by sonication in a 4 °C water bath. This homogenization cycle was repeated three times. After incubation at −40 °C for 1 h to precipitate proteins, samples were centrifuged at 13,800× *g* for 15 min at 4 °C. The supernatant was transferred to a fresh glass vial for analysis. Equal aliquots of supernatant from each sample were pooled to create a composite quality control (QC) sample. One QC sample was analyzed for every six experimental samples to monitor system stability and reproducibility [28].

#### 2.6.2. LC-MS/MS Analysis

Metabolomic profiling was conducted on a Vanquish UHPLC system (Thermo Fisher Scientific, Waltham, MA, USA) coupled to an Orbitrap Exploris 120 mass spectrometer (Thermo Fisher Scientific, Waltham, MA, USA). Chromatographic separation was achieved using a Waters ACQUITY UPLC BEH Amide column (2.1 mm × 50 mm, 1.7 μm). The mobile phase consisted of (A) 25 mM ammonium acetate and 25 mM ammonium hydroxide in water (pH 9.75) and (B) acetonitrile. The gradient elution program was as follows: 0–0.25 min, 5% A; 0.25–3.5 min, 5–35% A; 3.5–4.0 min, 35–60% A; 4.0–4.5 min, 60% A; 4.5–4.55 min, 60–5% A; 4.55–6.0 min, 5% A, followed by a 2.0 min post-run equilibration. The flow rate was 0.3 mL/min, column temperature was 35 °C, injection volume was 2 μL, and the auto-sampler was maintained at 4 °C. Mass spectrometric detection was performed in both positive and negative ionization modes with an *m*/*z* range of 70–1050 [43].

#### 2.6.3. Data Processing

Multivariate statistical analysis was performed using the ropls package (version 1.6.2) in R, including principal component analysis (PCA) and orthogonal partial least squares-discriminant analysis (OPLS-DA) with 7-fold cross-validation for model assessment. Univariate analysis (Student’s *t*-test) was also conducted. Metabolites with a Variable importance in projection (VIP) score > 1.0 and a *p* < 0.05 were considered significantly differential. These metabolites were subsequently mapped to the KEGG database (https://www.kegg.jp/kegg/pathway.html, accessed on 6 June 2025) for pathway enrichment analysis, with statistical significance being assessed using Fisher’s exact test [44].

### 2.7. Controlled SEM-Generating Reactions

NaClO (1000 mg/L, 1 mL), urea standard (10 mg/mL, 1 mL), lecithin (0.1 g), triglyceride (1 mL), Pro (10 mg), and Arg (10 mg) were mixed in nine different ways. The pH of each mixture was adjusted with 1 M HCl or NaOH, followed by incubation at room temperature (25 °C) for 12 h. The SEM concentration was determined as described in Section 2.3. Comparison of the SEM generated informed the investigation of SEM formation pathways [7].

### 2.8. Statistical Analyses

Quantitative data from three independent biological replicates are expressed as mean ± standard deviation. Statistical significance was evaluated using one-way ANOVA followed by Tukey’s test for multiple comparisons in SPSS Statistics 26.0 (IBM Corp., Armonk, NY, USA), with a significance threshold of *p* < 0.05. All figures were generated using Origin 2024 software (OriginLab, Northampton, MA, USA).

## 3. Results and Discussion

### 3.1. Effects of NaClO Bleaching Conditions on SEM Generation

**Figure 1:**
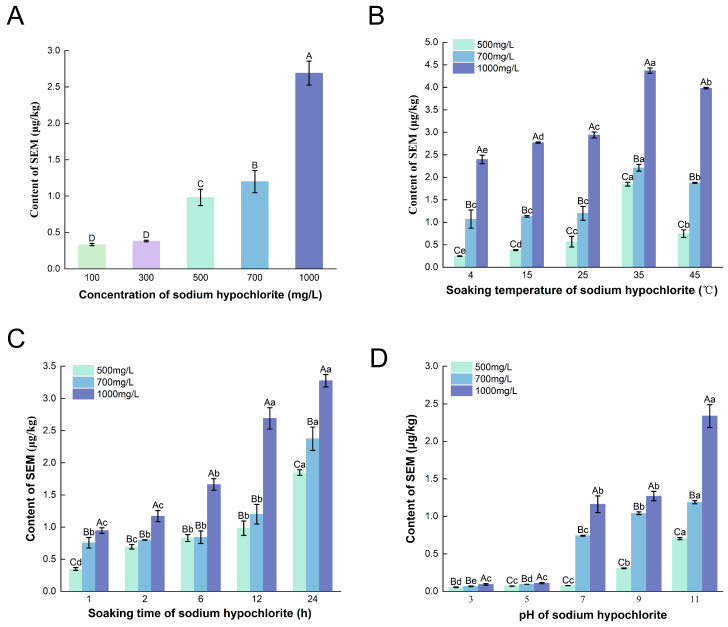
Effect of NaClO bleaching treatments on semicarbazide (SEM) content in basa (*Pangasius bocourti*) fish maw (BFM). (**A**) Effect of sodium hypochlorite (NaClO) concentration; (**B**) effect of soaking temperature; (**C**) rffect of soaking time; (**D**) rffect of NaClO solution pH. Notes: Different capital letters indicate significant differences between different NaClO concentrations (*p* < 0.05). Different lowercase letters indicate significant differences between treatments at the same concentration of NaClO (*p* < 0.05).

SEM generated under various bleaching conditions is illustrated in Figure 1. As available chlorine concentration increased (provided by the NaClO), SEM concentration in the BFM increased significantly, indicating that concentration of NaClO substantially impacted SEM formation (Figure 1A). When available chlorine increased from 100 to 1000 mg/L, SEM rose from 0.33 ± 0.02 μg/kg to 2.69 ± 0.17 μg/kg.

Immersion temperature, duration, and pH also significantly influenced SEM formation in BFM. As temperature increased, so did SEM concentration: low temperatures produced less SEM, but temperatures exceeding 25 °C stimulated a rapid increase. For NaClO solutions with available chlorine concentrations of 500, 700, and 1000 mg/L, SEM concentrations increased from 0.25, 1.07, and 2.40 μg/kg at 4 °C to 0.74, 1.88, and 3.98 μg/kg at 45 °C, respectively (Figure 1B).

Extending the immersion time produced a gradual increase in SEM (Figure 1C). For NaClO solutions with available chlorine concentrations of 500, 700, and 1000 mg/L, SEM concentrations increased from 0.35, 0.76, and 0.95 μg/kg at 1 h to 0.98, 1.20, and 2.69 μg/kg at 12 h, respectively. When immersion time was less than 12 h, SEM increased significantly in the 1000 mg/L available chlorine group, but no change was observed in the 500 and 700 mg/L groups. However, when immersion time was extended to 24 h, SEM increased further to 1.85, 2.37, and 3.27 μg/kg, respectively. A notable divergence was observed after 12 h: while SEM concentrations in the 1000 mg/L group plateaued, they continued to rise markedly in the 500 and 700 mg/L groups. This indicates that short-term immersion (<12 h) had a greater impact on SEM formation in BFM treated with high available chlorine concentrations (>700 mg/L, resulting in a 2.8-fold increase in SEM), while the effect was smaller when available chlorine was lower (<700 mg/L). The lack of further SEM formation in the highly available chlorine group after prolonged immersion (>12 h) may be attributed to the faster decomposition of NaClO during extended storage at room temperature, whereas the chlorine available in the lower-concentration solutions remained more stable [45].

Increasing pH of the bleaching solution also significantly enhanced SEM formation, with a particularly notable increase when pH exceeded 9 (Figure 1D). With available chlorine concentrations of 500, 700, and 1000 mg/L, SEM concentrations increased from 0.05, 0.07, and 0.09 μg/kg at pH 3 to 0.70, 1.19, and 2.34 μg/kg at pH 11, respectively.

Previous studies suggest that azines generated from natural food compounds can react with hydrogen peroxide, hypochlorite, or oxygen to form hydrazines, which subsequently react with urea-like compounds to form SEM [11]. In dairy processing, sodium hydroxide is used to adjust pH to prevent spoilage; however, localized high pH conditions (>10) can lead to significant increases in SEM [35]. According to Bendall [35], high pH favors SEM formation primarily through enhanced generation of the intermediate aminocyanic acid, which reacts with urea to form SEM. pH also affects the stability of available chlorine in NaClO solutions [46]. At lower pH, NaClO tends to decompose more readily, which enhances its sporicidal efficiency but shortens its effective shelf life. When the pH falls below 8.5, the shelf life of hypochlorite solutions decreases exponentially [47]. By contrast, higher pH maintains the activity of available chlorine, which may explain the increased SEM formation under high pH conditions observed in this experiment.

### 3.2. Physicochemical Properties of BFM

#### 3.2.1. BFM Texture Profile Analysis

**Figure 2:**
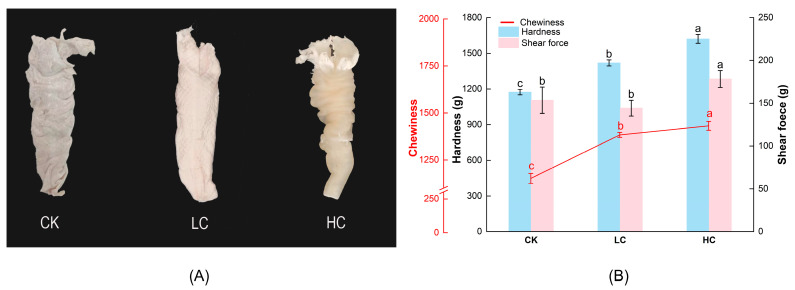
The morphological and textural properties of basa (*Pangasius bocourti*) fish maw (BFM) under different processing conditions. (**A**) Pictures of BFM. (**B**) Various bleaching treatments on the textural properties of BFM. Notes: CK: fresh BFM; LC: BFM treated with 100 mg/L sodium hypochlorite (NaClO); HC: BFM treated with 1000 mg/L NaClO. Different letters indicate significant differences (*p* < 0.05).

Texture is widely used as an indicator of food quality and mouthfeel, influencing consumer preference and acceptance [48]. To evaluate the impact of NaClO bleaching, the hardness, shear force, and chewiness of BFM samples (Figure 2A) were measured. All three parameters were affected by NaClO treatment. The HC group exhibited significantly enhanced hardness (1621.28 ± 37.39 g), chewiness (1431.44 ± 23.22), and shear force (178.32 ± 9.83 g), indicating that high-concentration NaClO substantially altered BFM texture compared to the CK control (hardness: 1173.62 ± 22.99 g, chewiness: 1151.84 ± 26.57, and shear force: 153.57 ± 15.35 g). The LC treatment did not change sample shear force (144.30 ± 9.25 g), but increased BFM hardness (1418.71 ± 25.35 g) and chewiness (1383.48 ± 13.30) (Figure 2B).

Consistent with the findings of the present study, Fu et al. [31] also observed that tilapia fillets treated with NaClO exhibited higher springiness, cohesiveness, hardness, adhesiveness, and overall textural parameters compared to the control group, indicating that the oxidative effect of sodium hypochlorite induced protein denaturation, leading to structural changes, which in turn resulted in cross-linking and aggregation, and consequently increased textural parameters. Xia et al. [49] reported that moderate oxidation (10 mM H_2_O_2_) enhanced the hardness and elasticity of myofibrillar protein gels. Additionally, Wang et al. [7] observed that increasing H_2_O_2_ concentrations resulted in a decrease in the shear force and elasticity of squid meat. However, subsequent phosphate treatment after H_2_O_2_ oxidation was found to improve the textural properties of the squid meat. This improvement is attributed to structural changes in myofibrillar proteins induced by oxidation, which promote the formation of new protein cross-links. Phosphate treatment may help maintain this oxidative cross-linking at a relatively optimal level. Conversely, excessive oxidation may lead to severe disruption and aggregation of myofibrils, thereby adversely affecting the texture of squid meat. Collectively, these findings indicate that oxidative treatment induces structural changes in myofibrillar proteins, consequently leading to alterations in the textural properties of the final product [50]. In the present study, the observed significant increases in hardness, chewiness, and springiness of BFM with increasing NaClO concentration may be attributed to the fact that, at these concentrations, NaClO induced structural changes in myofibrillar proteins at an optimal level without causing severe disruption of the myofibrils, indicating that moderate protein oxidation may be an effective approach to improving the texture of BFM.

#### 3.2.2. Microstructure Analysis

**Figure 3:**
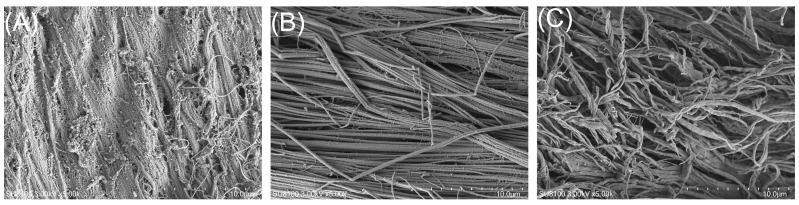
Scanning electron microscopy images of basa (*Pangasius bocourti*) fish maw (BFM). (**A**) CK: fresh BFM (×5000); (**B**) LC: BFM treated with 100 mg/L sodium hypochlorite (NaClO) (×5000); (**C**) HC: BFM treated with 1000 mg/L NaClO (×5000).

Scanning electron microscopy was used to verify the impact of NaClO bleaching on fibrillar proteins in BFM by comparing the microstructures of the samples (Figure 3). NaClO bleaching induced marked structural changes in the fibrillar proteins of BFM. Untreated BFM (CK; Figure 3A) exhibited an orderly arrangement of myofibrils. The fibers in the LC group (Figure 3B) largely retained this ordered structure, although localized fiber bending and the initial formation of small bundles were observed. The HC group (Figure 3C) showed pronounced distortion of the myofibrils, which aggregated extensively into bundles [51].

This is consistent with the microstructural changes in oxidized squid meat reported by Wang et al. [7]. In the non-oxidized group, the myofibrils were loosely and orderly arranged, with distinct gaps between the fibers. As the degree of oxidation increased, the muscle fibers tended to aggregate, and the myofibrillar bundle structure became more compact. Honeycomb-like structural characteristics with smaller internal pores provide the swim bladder with greater resistance to deformation, giving it a stiffer texture and better elasticity and chewiness [52]. This is consistent with the findings of Jiang et al. [53], who described squid muscle fibers as loose and orderly with distinct gaps, whilst oxidation caused significant aggregation of the fibers [54]. NaClO treatment transformed the internal structure of the BFM from tightly arranged fiber bundles into a porous network, endowing it with superior deformation recovery capacity [55,56] and enhancing its elasticity and chewiness.

### 3.3. Collagen Physicochemical Properties

#### 3.3.1. Collagen Concentration

The concentrations of Hyp and collagen did not differ significantly between the LC and CK groups. However, the HC group exhibited reductions in both concentrations (*p* < 0.05), indicating that high-concentration NaClO caused a degree of collagen loss in BFM (Table 1).

Previous studies show that collagen with an intact triple-helix structure is almost insoluble in water, but it becomes soluble when degraded into collagen peptides. Since Hyp is a characteristic amino acid of collagen [57], the release of water-soluble Hyp indicates collagen degradation. The swelling of collagen fibers under acidic or alkaline conditions is related to the osmotic pressure difference between the protein phase and the external solution (as well as electrostatic repulsion) [58]. pH has a major influence on collagen fiber swelling, with maximum swelling occurring at pH 2.2 and 11.8. pH may also affect collagen solubility via altered intermolecular interactions and molecular conformation. Wolf, et al. [59] applied different pH conditions to bovine hide collagen powder and found that collagen solubility varied between 28.9% and 52.5%, with the highest solubility occurring at pH 2.0 and 12.0 and the lowest around pH 8.0. This is consistent with the present findings, where increased pH led to higher solubility and loss of collagen during the rinsing of BFM.

#### 3.3.2. SDS-PAGE

**Figure 4:**
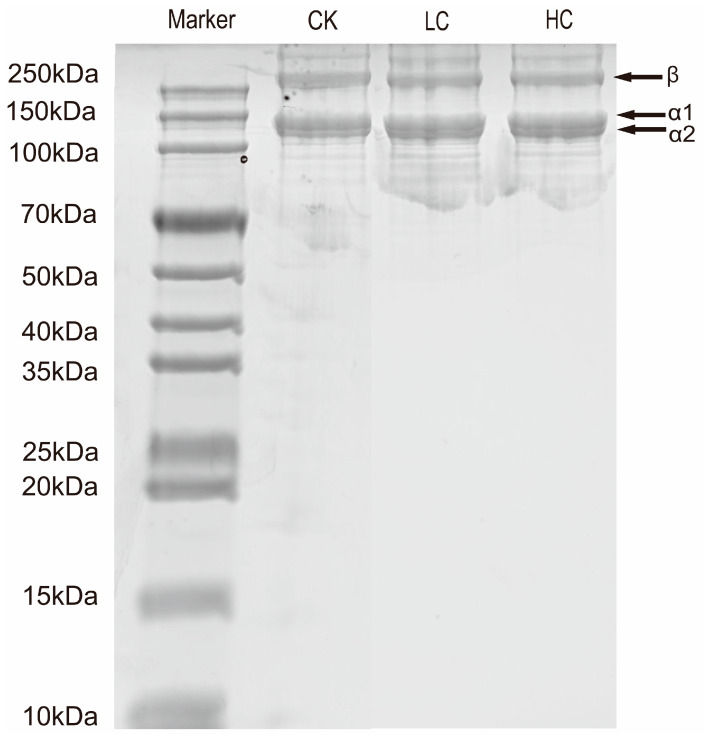
SDS-PAGE analysis of collagen extracted from basa (*Pangasius bocourti*) fish maw (BFM). Notes: Marker: standard protein marker; CK: collagen from fresh BFM; LC: collagen from BFM treated with 100 mg/L sodium hypochlorite (NaClO); HC: collagen from BFM treated with 1000 mg/L NaClO.

Collagen exhibits a fibrous structure and higher solubility in acidic media. Consequently, acidic solutions (e.g., 0.5 M acetic acid) are commonly used in collagen extraction, yielding acid-soluble collagen [38]. SDS-PAGE revealed that acid-soluble swim bladder collagen largely retained the native structure of the macromolecule (Figure 4). Distinct bands were observed above 180 kDa, indicating the presence of γ-chains (trimers of α-chains) and β-chains (dimers of α-chains), which suggests a high degree of cross-linking in BFM collagen. α-chains, including α1 (130 kDa) and α2 (110 kDa), were clearly evident in the 135–180 kDa region. This indicates that the structural characteristics of acid-soluble swim bladder collagen were similar to those of type I collagen, with high extraction purity and no apparent contaminating bands. Type I collagen (found primarily in connective tissues such as skin, bone, and tendons) consists of one α1 chain and one α2 chain [60]. It is uniquely characterized by a right-handed triple superhelix structure formed by three α-chains of similar size [61].

The collagen α-chain bands in the LC and HC groups exhibited a degree of smearing. This is consistent with the findings of Nagarajan et al. [57], who observed a decrease in the intensity of the α-chain bands and the disappearance of low-molecular-weight protein bands (70 and 76 kDa) in gelatin extracted from bleached squid skin, indicating that strong oxidizing agents such as H_2_O_2_ may induce cleavage of the α-chains and lead to the loss of certain low molecular weight proteins. Similar results have also been reported by Aewsiri et al. [62] and Hoque et al. [63] in gelatin extracted from bleached cuttlefish skin.

#### 3.3.3. FTIR Analysis

**Figure 5:**
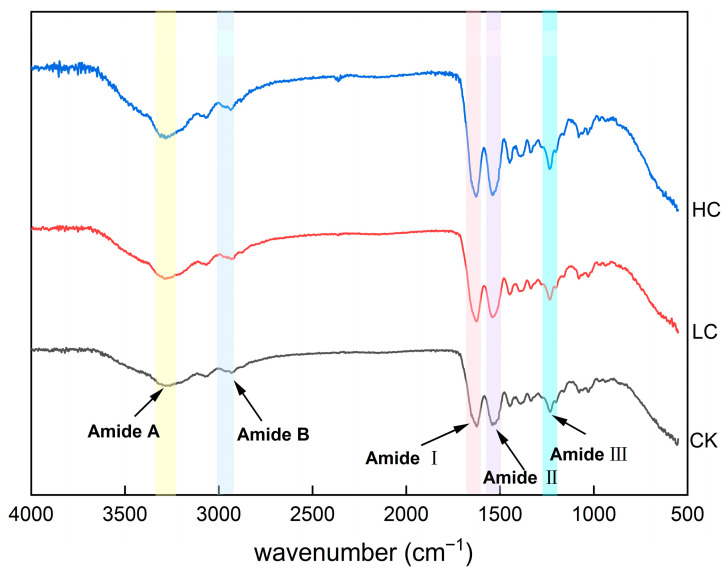
FTIR spectra of collagen extracted from basa (*Pangasius bocourti*) fish maw (BFM). Notes: CK: collagen from fresh BFM; LC: collagen from BFM treated with 100 mg/L sodium hypochlorite (NaClO); HC: collagen from BFM treated with 1000 mg/L NaClO.

BFM is rich in proteins, with collagen being the most important structural protein [64]. Tropocollagen, the fundamental unit of collagen, consists of three polypeptide chains that coil around each other in a stable triple-helix structure held by intermolecular hydrogen bonds [65]. In aquatic products, changes in proteins often lead to changes in microstructure [66]. FTIR spectra of BFM were similar across the bleaching treatments, each exhibiting typical amide I, II, and III bands and amide A and B bands (Figure 5).

Amide A bands were observed at 3259.21, 3286.37, and 3283.92 cm^−1^ in the CK, LC, and HC groups, respectively. This band originates from the stretching vibration of N–H groups coupled with hydrogen bonding [67]. Typically, stretching vibrations of free N–H groups occur in the 3400–3440 cm^−1^ range. When N–H groups in peptide chains participate in hydrogen bonding, the vibration peak shifts to a lower wavenumber, usually around 3300 cm^−1^ [68]. Notably, HC exhibited a higher amplitude Amide A band than the other groups, which might be due to the generation of free amino groups arising from collagen degradation. Amide B bands in CK, LC, and HC samples were observed at 2927.90, 2933.46, and 2937.055 cm^−1^, respectively. This band is associated with the asymmetric stretching vibration of C–H in –CH_2_ groups [69].

The Amide I, II, and III bands reflect the conformation of the protein polypeptide chain. Amide I absorption bands were observed at 1624.03, 1624.46, and 1628.38 cm^−1^ in the CK, LC, and HC groups, respectively. This band is associated with the coupling of C=O stretching vibrations and C–N stretching vibrations [68]. The integrity of the collagen triple helix can be assessed from the Amide I band due to hydrogen bonding of C=O with adjacent groups [69]. The shift in the Amide I band to a higher wavenumber in the HC group might be related to its reduced α-chain content. Amide II bands resulting from C–N stretching or N–H bending vibrations were observed in CK, LC, and HC samples in the wavenumber range 1540–1543 cm^−1^ [70]. The Amide III band—another characteristic infrared absorption peak for collagen—was observed around 1232–1235 cm^−1^ in all BFM samples. This is associated with C–N stretching vibrations and N–H deformation induced by amide bonds, arising from the wagging vibration of –CH_2_ groups in the glycine backbone and Pro side chains of collagen [42]. The large Amide III peak amplitude in HC group samples may be due to alkaline swelling following NaClO treatment, leading to exposure of the collagen tertiary structure [71]. This is consistent with Xu et al. [58], who investigated the secondary structure of collagen under different pH conditions and found that acid swelling promoted exposure of the collagen triple helix structure, increasing the ratio of the Amide III to 1450 cm^−1^ bands (AIII/A1450). Thus, bleaching affected the secondary structure and functional group characteristics of collagen in BFM.

### 3.4. Untargeted Metabolomics

**Figure 6:**
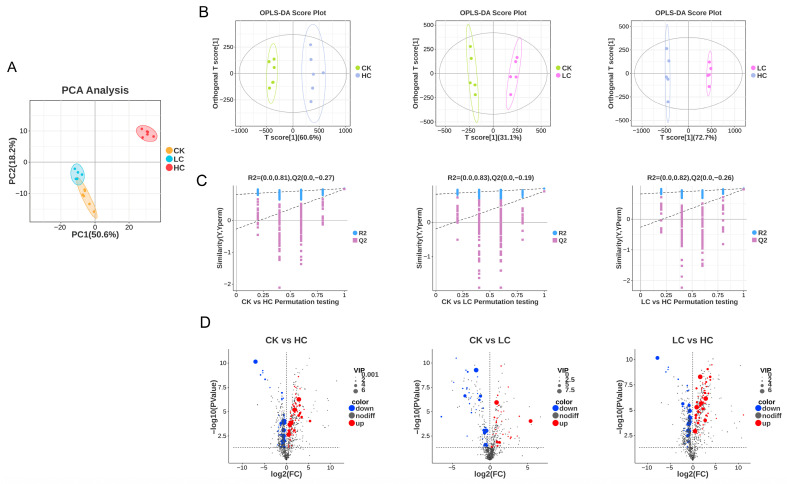
(**A**) Principal component analysis (PCA) scores plot of CK, LC and HC; (**B**) orthogonal partial least squares-discriminant analysis (OPLS-DA) score plots of CK, LC and HC; (**C**) OPLS-DA permutation test plots of CK, LC and HC; (**D**) differential statistics and volcano maps of CK, LC and HC. Notes: CK: fresh basa (*Pangasius bocourti*) fish maw (BFM); LC: BFM treated with 100 mg/L sodium hypochlorite (NaClO); HC: BFM treated with 1000 mg/L NaClO.

PCA is a statistical method for analyzing multivariate data using unsupervised pattern recognition [72]. The principal components, which explain the largest proportion of variance in the data matrix, are derived from the original variables, retaining as much information as possible while remaining uncorrelated with each other. PCA was used to provide an overall assessment of the samples from the three groups. Clear separation of the CK and HC samples was evident in both positive and negative ionization modes (Figure 6A), indicating significant inter-group differences and that metabolites were substantially affected by bleaching. By contrast, no clear separation of the CK and LC groups was evident, suggesting their metabolic profiles were similar. The proximity of data points within each group demonstrated the low variability and consistent pattern of metabolites present in BFM samples subjected to the same treatment.

OPLS-DA was also applied to the metabolomics data. The results demonstrated clear separation of groups in the OPLS-DA score plots, good intra-group reproducibility, and stable, reliable models (Figure 6B). In the OPLS-DA model validation plots, the abscissa denotes permutation retention, while the ordinate displays the corresponding R^2^ (blue circles) and Q^2^ (purple squares) values from permutation tests, and regression lines for R^2^ and Q^2^ are indicated by dashed lines. The proximity of R^2^ to Q^2^, with both values being close to 1, indicates stable and reliable models (Figure 6C).

#### 3.4.1. Differential Metabolite Analysis

A volcano plot visualizes the distribution and changing trends of differential metabolites between two sample groups. Metabolites exhibiting a fold change (FC) above a threshold of 1 were selected to create the differential volcano plots shown in Figure 6D. The *x*-axis displays the log2(FC) in metabolite abundance between the two groups, and the *y*-axis displays the statistical significance of the difference in abundance, expressed as −log10(*p*-value), with higher values indicating greater significance. Each datapoint in a plot represents a specific metabolite, with the point size corresponding to the VIP value. Red datapoints denote significantly up-regulated metabolites, blue points denote significantly down-regulated metabolites, and grey points denote unchanged metabolites (Figure 6D).

**Figure 7:**
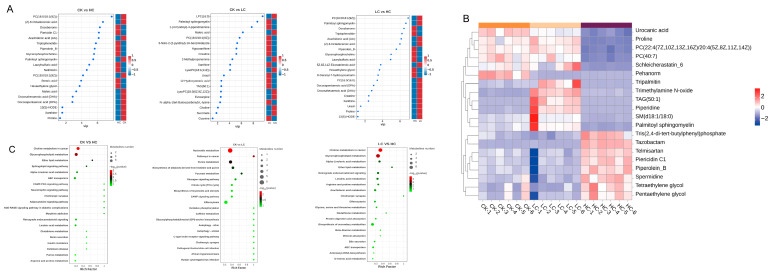
(**A**) VIP value analysis of CK, LC and HC; (**B**) metabolite clustering analysis of CK, LC and HC; (**C**) KEGG analysis of CK, LC and HC. Notes: CK: fresh basa (*Pangasius bocourti*) fish maw (BFM); LC: BFM treated with 100 mg/L sodium hypochlorite (NaClO); HC: BFM treated with 1000 mg/L NaClO.

Using OPLS-DA data, differential metabolites were screened using the criteria VIP > 1, *p* < 0.05, and log2(FC) > 1 or <−1 (Figure 7A) [73]. VIP > 1 was used to identify metabolites that made significant contributions to the separation between the two groups [74]; *p* < 0.05 was applied to select metabolites with statistical significance; and log2(FC) > 1 or <−1 was employed to ensure a sufficient magnitude of change. Specifically, a log2(FC) > 1 indicates a more than two-fold up-regulation, whereas a log2(FC) < −1 indicates a more than two-fold down-regulation [24,44]. Preliminary screening identified 72 potential differential metabolites in ESI+ mode and 40 in ESI- mode. HC exhibited 62 up-regulated and 46 down-regulated metabolites compared to the CK group, while LC exhibited 35 up-regulated and 35 down-regulated metabolites, and LC exhibited 57 up-regulated and 41 down-regulated metabolites. The main types of differential metabolites were: fatty acids and conjugates, amino acids, peptides and analogues, benzoic acids and derivatives, carbohydrates and conjugates, eicosanoids, amines, alcohols and polyols, and purines and purine derivatives. This demonstrates the breadth of changes in the metabolic profile of BFM due to bleaching.

The top 20 differential metabolites were selected for comparative analysis (Figure 7B). These metabolites are considered key contributors to the metabolic alterations in BFM induced by NaClO treatment. NaClO is an oxidative bleaching agent that induces oxidative stress in lipids, amino acids, etc. This study shows that oxidative stress intensifies with increasing NaClO concentration. Proteins are primary targets for hypochlorite [75]. Chloramines formed from the reaction of amino groups with hypochlorite generate free radicals that induce lipid peroxidation [76,77]. This is consistent with the changes observed in the lipids and related compounds in the HC group (Figure 7B). Hypochlorite can react with DNA, RNA, and polynucleotides to produce various nitrogen-based radicals. Experiments with mixtures of nucleotides indicate that hypochlorite tends to form radicals with polynucleotides following the order: cytidine > adenosine = guanosine > uridine = thymidine [78]. The increased levels of xanthine in the LC treatment group might have resulted from nucleic acid oxidative damage by NaClO (Figure 7A).

#### 3.4.2. KEGG Pathway Enrichment Analysis

A collection of functionally interconnected metabolites constitutes a metabolic pathway. Variations in the output of a pathway reflect the cumulative differences in expression of the metabolites within it. Pathway enrichment analysis calculated the enrichment *p*-value for each pathway associated with each differential metabolite using the KEGG database. Important metabolic pathways that were affected by the experimental intervention were inferred from the pathways that were significantly enriched [79,80].

Nine significantly enriched pathways were evident in the HC group compared to the CK control group (Figure 7C). These included Lipid Metabolism pathways such as biosynthesis of unsaturated fatty acids, linoleic acid metabolism, and glycerophospholipid metabolism, as well as Organismal System- and Human Disease-related pathways. LC exhibited five enriched pathways compared to CK: nucleotide metabolism, purine metabolism, biosynthesis of histidine and purine-derived alkaloids, pyruvate metabolism, and pathways in cancer. A comparison of LC and HC yielded seven enriched pathways: biosynthesis of unsaturated fatty acids, choline metabolism in cancer, glycerophospholipid metabolism, retrograde endocannabinoid signaling, linoleic acid metabolism, beta-alanine metabolism, and ether lipid metabolism. It can be concluded that lipid, purine, and nucleotide metabolisms are the main differential metabolic pathways when BFM is exposed to NaClO bleaching. The strong oxidizing effect of NaClO on lipid metabolism in BFM was clearly evident (Figure 7C), although the generation of SEM via oxidation of lipids by NaClO requires further experimental validation.

KEGG pathway enrichment analysis showed that NaClO treatment also altered Arg and Pro metabolism in BFM (Figure 7C). Urocanic acid and Pro contents decreased considerably following HC treatment compared to the CK and LC groups (Figure 7B), suggesting that NaClO promoted degradation of urocanic acid and metabolism of Pro. Degradation of urocanic acid provides a nitrogen source for the urea cycle [81]. Pro metabolism and synthesis are linked to the tricarboxylic acid cycle, urea cycle, and pentose phosphate pathway. Pro metabolism also generates glutamate and NH_3_, causing accumulation of nitrogenous compounds [82]. Ammonia nitrogen that an organism cannot excrete may be metabolically converted to SEM under the strong oxidative effect of NaClO. Evidence indicates that aquatic products are rich in nitrogenous compounds containing guanidino or ureido groups, such as Arg, His, citrulline, and creatinine. These compounds contain structures similar to SEM. It is therefore hypothesized that such nitrogenous compounds undergo degradation reactions when exposed to hypochlorite solutions, releasing SEM [10]. The abundance of Pro in collagen and the high collagen content of fish maw mean that Pro metabolism will have a significant influence on SEM formation in this product. SEM formation during NaClO bleaching may occur through reactions between hypochlorite and N-containing compounds. One possible pathway is analogous to the Hofmann reaction, which has been reported to produce SEM [35].

### 3.5. Controlled SEM-Generating Reactions

**Figure 8:**
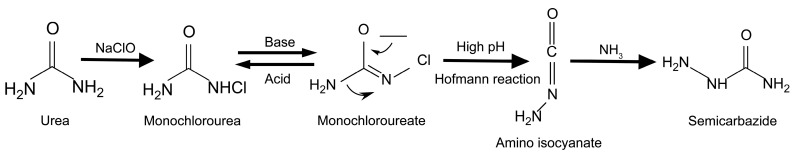
Formation of semicarbazide (SEM) via the Hofmann reaction.

To verify the metabolomics findings and elucidate the mechanism by which NaClO induces SEM generation in BFM, a controlled oxidation experiment was conducted (Table 2). No SEM formation was detectable from urea or NaClO alone under alkaline conditions. However, when NaClO and urea were mixed, SEM was generated, with considerably more being produced under alkaline conditions (41.29 ± 0.02 μg/kg at pH 11) than at neutral pH (1.28 ± 0.03 μg/kg at pH 7). No SEM was detected when urea or NaClO was mixed with lecithin or triglycerides. By contrast, when urea, NaClO, lecithin, and triglycerides were mixed, the SEM concentration was 1.16 ± 0.11 μg/kg, which was not significantly different from the NaClO–urea mixture. Mixing NaClO with Pro or Arg yielded small amounts of SEM (0.254 ± 0.06 and 0.311 ± 0.04 μg/kg, respectively). These findings indicate that the generation of SEM in BFM following NaClO application was primarily due to the Hofmann reaction between the hypochlorite and N-containing substances such as urea [35] (Figure 8). This is consistent with the findings of Bendall [35]. Lecithin and triglyceride had no impact on SEM formation.

## 4. Conclusions

This study demonstrates that whileNaClO bleaching improves the texture of BFM by promoting fibrillar protein aggregation, it also disrupts collagen structure, reduces collagen content, and induces SEM formation. SEM generation was found to correlate positively with chlorine concentration, bleaching duration, temperature, and pH, with alkaline conditions markedly enhancing its formation. These findings underscore the need for stringent control of processing parameters—especially pH—to mitigate SEM accumulation. Mechanistic insights from untargeted metabolomics suggest that SEM may originate from Hofmann reactions between NaClO and nitrogenous compounds. This is the first study to link NaClO bleaching with both textural changes and SEM formation in BFM, offering a scientific basis for improving product safety. Given the exploratory nature of untargeted metabolomics, future studies should employ targeted approaches such as isotope-labeled tracer techniques to validate the proposed pathways. Additionally, exploring non-oxidizing bleaching agents like sodium sulfite may provide safer alternatives to NaClO, thereby supporting the sustainable development of the fish maw processing industry.

## Figures and Tables

**Table 1:** Effect of sodium hypochlorite (NaClO) treatment on hydroxyproline (Hyp) and collagen content in basa (*Pangasius bocourti*) fish maw (BFM).

Group	Hyp Content (μg/g)	Collagen Content (μg/g)
CK	6.92 ± 0.08 a	49.44 ± 0.57 a
LC	6.86 ± 0.08 a	49.01 ± 0.57 a
HC	5.62 ± 0.22 b	40.11 ± 1.57 b

Notes: CK: fresh BFM; LC: BFM treated with 100 mg/L NaClO; HC: BFM treated with 1000 mg/L NaClO. Data are expressed as mean ± standard deviation. Different letters in the same column indicate significant differences among the groups (*p* < 0.05).

**Table 2:** Semicarbazide (SEM) content of standardized compounds with different combinations.

Sample	Content of SEM (μg/kg)
NaClO (pH = 11)	ND
Urea (pH = 11)	ND
NaClO + Urea (pH = 7)	1.28 ± 0.03
NaClO + Urea (pH = 11)	41.29 ± 0.02
NaClO + Pro	0.254 ± 0.06
NaClO + Arg	0.311 ± 0.04
NaClO + Lecithin + Triglyceride (pH = 7)	ND
NaClO + Urea + Lecithin + Triglyceride (pH = 7)	1.16 ± 0.11
Urea + Lecithin + Triglyceride (pH = 7)	ND

Data are expressed as mean ± standard deviation. ND: not detected.

## Data Availability

The original contributions presented in this study are included in the article. Further inquiries can be directed to the corresponding authors.

## Abbreviations

The following abbreviations are used in this manuscript:

BFM	Basa (*Pangasius bocourti*) fish maw
SEM	Semicarbazide
VIP	Variable Importance in Projection
PCA	Principal Component Analysis
OPLS-DA	Orthogonal Partial Least Squares-Discriminant Analysis
